# Modeling Heterogeneous Brain Dynamics of Depression and Melancholia Using Energy Landscape Analysis

**DOI:** 10.3389/fpsyt.2021.780997

**Published:** 2021-11-25

**Authors:** Paul Rossener Regonia, Masahiro Takamura, Takashi Nakano, Naho Ichikawa, Alan Fermin, Go Okada, Yasumasa Okamoto, Shigeto Yamawaki, Kazushi Ikeda, Junichiro Yoshimoto

**Affiliations:** ^1^Division of Information Science, Graduate School of Science and Technology, Nara Institute of Science and Technology, Ikoma, Japan; ^2^Department of Computer Science, College of Engineering, University of the Philippines Diliman, Quezon City, Philippines; ^3^Center for Brain, Mind and KANSEI Research Sciences, Hiroshima University, Hiroshima, Japan; ^4^Department of Neurology, Faculty of Medicine, Shimane University, Izumo, Japan; ^5^School of Medicine, Fujita Health University, Toyoake, Japan; ^6^Department of Psychiatry and Neurosciences, Hiroshima University, Hiroshima, Japan

**Keywords:** depression, melancholia, energy landscape analysis, resting state fMRI, functional brain network

## Abstract

Our current understanding of melancholic depression is shaped by its position in the depression spectrum. The lack of consensus on how it should be treated—whether as a subtype of depression, or as a distinct disorder altogethe—interferes with the recovery of suffering patients. In this study, we analyzed brain state energy landscape models of melancholic depression, in contrast to healthy and non-melancholic energy landscapes. Our analyses showed significant group differences on basin energy, basin frequency, and transition dynamics in several functional brain networks such as basal ganglia, dorsal default mode, and left executive control networks. Furthermore, we found evidences suggesting the connection between energy landscape characteristics (basin characteristics) and depressive symptom scores (BDI-II and SHAPS). These results indicate that melancholic depression is distinguishable from its non-melancholic counterpart, not only in terms of depression severity, but also in brain dynamics.

## 1. Introduction

Depression and melancholia are synonymous terms in common speech, but have different technical meanings in psychiatry (1). While depression is associated with “deepened or prolonged sadness in everyday life,” melancholia is predominantly marked by “loss of pleasure or interest” (or anhedonia), along with many other depressive symptoms (1, 2). In the Diagnostic and Statistical Manual of Mental Disorders, third edition (DSM-III), depression and melancholia were clumped together into a single disorder: the major depressive disorder (MDD) (3). However, this has been rectified in DSM-IV, albeit not entirely, by recognizing melancholia as a specifier for depression (4). The recognition has attracted research on melancholic depression as a distinct subtype of depression, which has been neglected before in clinical assessment and treatment (2, 5, 6).

Since then, there has been significant thrust on classifying melancholic depression. Because of the overlapping symptoms between depression and melancholia, researchers have been focusing on distinguishing the two. This distinction is important for both diagnosis and treatment, since melancholic patients (i.e., MDD with melancholic features) may have different responses to treatment (2). For example, they respond better to tricyclic antidepressants (TCAS) than to selective serotonin reuptake inhibitors (SSRIs) (7). On the contrary, they do not respond well with psychotherapy and placebo, as compared to non-melancholic patients (8). Thus clearly, correct diagnosis of melancholic depression is the first step toward the right treatment.

In the recent years, computational and statistical models have been used to differentiate melancholic from non-melancholic depression (9). The complexity and design of these models range from fundamental statistical analysis (10–13) to machine learning models (14–16). In most of these studies, they analyzed neuroimaging data to identify the brain activities involved in melancholic depression (11–16). In particular, they extracted functional connections from resting state functional MRI (rs-fMRI) data (13–16). Functional connectivity analysis (FCA) provides insights on which brain regions are temporally correlated, and is useful for studying brain networks and neural circuit dynamics (14). However, FCA-based models assume that pairwise interactions between brain regions are independent of each other, and thus, may disregard information about higher-order interactions (17).

Our research further explores the brain dynamics of melancholic depression. Several neuropsychiatric disorders are rooted in cognitive dysfunctions caused by disruptions in the dynamics of functional brain networks (18, 19). Evidences from previous FCA studies show that functional disconnections are prevalent in melancholic depression (13, 14). Here, we use energy landscape analysis (ELA) as an alternative to FCA. Embedded in ELA is the pairwise maximum entropy model (P-MEM), which estimates both individual activities and pairwise interactions of brain regions (17). Compared to FCA, P-MEM has been shown to be more physiologically informative since it infers global activity patterns instead of independent pairwise interactions. Furthermore, ELA accurately models the structure of multistable brain networks by reflecting both anatomical and functional connections in rs-fMRI data (17).

In ELA, the brain is characterized as a dynamic system that switches to multiple stable states (20). Each state is defined by its energy, which is inversely proportional to its probability of occurrence; thus, states with lower energy have higher probability. And since the states will vary in energy level, the energy landscape of these states can be portrayed by “peaks” (unstable) and “basins” (stable states), such that the brain has the tendency to “roll” down the basins. It would be interesting to see if this imagery of the brain being mired in a basin translates to the depressive feeling of being in a stuck state (21).

This study examines the energy landscapes of melancholic depression. Here, we show that melancholic depression exhibits a distinct energy landscape, in which its switching dynamics will be different from that of non-melancholic and healthy brain networks. By building an energy landscape model for melancholic depression, we further delineate the boundary between melancholia and depression; not necessarily to set them apart, but rather to clear the obfuscations that currently entangle them.

## 2. Materials and Methods

### 2.1. Study Participants

This study is a secondary analysis of rs-fMRI data of healthy and depressed volunteers recruited starting in 2012 from four medical institutions in Hiroshima, Japan (15). Prior to the administration of any experimental procedure, written informed consent was obtained from all participants. The experiments were carried out in accordance with relevant guidelines and regulations, and all our experimental protocols were approved by the Ethics Committee of Hiroshima University. The mental condition of the volunteers were evaluated using Mini International Neuropsychiatric Interview (M.I.N.I) according to the Diagnostic and Statistical Manual of Mental Disorders, 4th ed. (DSM-IV) criteria (22). Initial screening resulted to 281 healthy participants with no history of any mental or neurological disorder, and 281 diagnosed with major depressive disorder (MDD).

Additional screening of participants was performed to emphasize the boundary between groups in the data set. First, the participants were screened based on their Beck Depression Inventory-II scores (BDI-II; healthy ≤ 13; depressed ≥ 20) (23). Second, participants were removed if they have incomplete data during interview or excessive head movements during fMRI recording. Third, healthy female participants were randomly sampled to match the number of healthy males. Last, the depressed participants were categorized into the melancholic group if they exhibited melancholic features (M.I.N.I, DSM-IV criteria) (22). Otherwise, they are categorized into the non-melancholic group.

In the end, a total of 142 healthy (71 F / 71 M) and 120 depressed (60 F / 60 M) participants were screened and selected for analysis. Within the depressed group, there were 89 melancholic (44 F / 45 M) and 31 non-melancholic (16 F / 15 M) participants. Details on their demographic data are summarized in Table 1.

**Table 1:** Demographic data of healthy and depressed participants.

		**Depressed**		
	**Healthy**	**Non-melancholic**	**Melancholic**	***P*-value**
No. of participants	142	31	89	
Sex (female/male)^*a*^	71 / 71	16 / 15	44 / 45	
Age (years)^*b*^	42.62 ± 14.33	41.48 ± 9.46	43.37 ± 11.65	
BDI-II^*b*^	5.34 ± 3.76	29.13 ± 6.08	32.40 ± 7.88	^(*)^ ^(***)^
Anhedonia (SHAPS)^*b*^	23.27 ± 0.66	34.21 ± 1.15	37.72 ± 0.66	^(*)^ ^(***)^
IQ (JART)^*b*^	110.92 ± 8.813	114.09 ± 9.57	111.61 ± 9.30	
Site participants (HUH/HRC/HKH/COI)^*a*^	44 / 32 / 20 / 46	10 / 6 / 7 / 8	48 / 7 / 13 / 21	^(**)^
Time samples per participant	161.90 ± 45.51	156.68 ± 50.34	155.39 ± 40.89	

*BDI-II, Beck Depression Inventory-II; JART, Japanese Adult Reading Test*.

*Recruitment sites: Hiroshima University Hospital (HUH), Hiroshima Rehabilitation Center (HRC), Hiroshima Kajikawa Hospital (HKH), Center of Innovation in Hiroshima University (COI)*.

a *Multiple group comparison using pairwise Chi-squared tests*.

b *Multiple group comparison using one-way ANOVA with Bonferroni correction*.

(*) *p < 0.05 between Non-melancholic and Melancholic groups*.

(**) *p < 0.01 between Healthy and Melancholic groups*.

(***) *p < 0.005 between Healthy and Non-melancholic groups, and between Healthy and Melancholic groups*.

### 2.2. Data Acquisition and Preprocessing

We recorded resting-state functional Magnetic Resonance Imaging (rs-fMRI) data of the participants using gradient echo planar imaging (EPI) sequences. The imaging device and parameters differ depending on the recording site (15). During recording, participants were instructed to focus on a cross mark displayed on a monitor, and to avoid thinking of anything or falling asleep. The fMRI recording lasted for 5–10 min, depending on the recording site (Table 2).

**Table 2:** Imaging protocols for different fMRI recording sites in Hiroshima.

	**HUH**	**HRC**	**HKH**	**COI**
MRI scanner	GE SignaHD x t	GE SignaHD x t	Siemens Spectra	Siemens Verio
Magnetic field strength (T)	3.0	3.0	3.0	3.0
Channels per coil	8	8	12	12
Field of view (mm)	256 x 256	256 x 256	192 x 192	212 x 212
Matrix	64 x 64	64 x 64	64 x 64	64 x 64
Number of slices	32	32	38	40
Number of volumes	143	143	107	240
In-plane resolution (mm)	4.0 x 4.0	4.0 x 4.0	3.0 x 3.0	3.3125 x 3.3125
Slice thickness (mm)	4.0	4.0	3.0	3.2
Slice gap (mm)	0.0	0.0	0.0	0.8
TR (ms)	2.0	2.0	2.7	2.5
TE (ms)	27.0	27.0	31.0	30.0
Total scan time (min)	5	5	5	10
Flip angle (deg)	90	90	90	80
Slice acquisition order	Ascending (interleaved)	Ascending (interleaved)	Ascending (interleaved)	Ascending

*HUH, Hiroshima University Hospital; HRC, Hiroshima Rehabilitation Center; HKH, Hiroshima Kajikawa Hospital; COI, Center of Innovation in Hiroshima University*.

Data preprocessing for the rs-fMRI data was performed using SPM8 (Wellcome Trust Centre for Neuroimaging, University College London, UK) on Matlab (Mathworks inc., USA). The preprocessing procedure included slice-timing correction, mean image realignment, normalization and resampling through segmentation of structural image aligned with the mean functional image, and smoothing with an isotropic 6 mm full-width half-maximum Gaussian kernel (24). The potential confounding effects (i.e., the temporal fluctuations of the white matter, cerebrospinal fluid, and the global signal, as well as six head motion parameters) were linearly regressed out from the fMRI time series to remove the physiological noise and motion artifacts (25, 26). These components were determined by the T1 images, which were simultaneously recorded with the rs-fMRI data. Finally, head motion artifacts were scrubbed from the functional images based on the relative changes (i.e., translational displacements along X, Y, and Z axes, and rotational displacements of pitch, yaw, and roll) between the image frames through time, with a frame-wise displacement (FD) threshold = 0.5 mm (27).

### 2.3. Functional Brain Networks

When selecting the regions of interest (ROIs) for analysis, we considered functional brain networks that were associated with depression. Time series data were extracted from 74 ROIs according to the 90-ROI Shirer Brain Atlas (28). Some ROIs were excluded due to either unreliable images during recording (e.g., in cerebellum area) (29), or insufficient number of ROIs of corresponding functional network (e.g., primary and higher visual networks). When extracting the time series data, the global mean and confounding effects of both cerebrospinal fluid (CSF) and white matter were linearly regressed out as part of the preprocessing step. A total of 12 distinct functional brain networks were analyzed in this study. For the brevity of this paper, we highlighted most of the results for three main networks: basal ganglia network (BGN), dorsal default mode network (DDMN), and left executive control network (LECN). These networks have been associated with melancholic depression (14, 30), and melancholic symptoms such as anhedonia (31) and rumination (32). The list of anatomical ROIs for all of the analyzed networks networks are listed in Table 3 and Supplementary Table 1.

**Table 3:** Functional brain networks associated with melancholic depression symptoms.

**Network**		**Anatomical locations of functional ROIs**	**Depressive symptoms**
Basal ganglia network (BGN)	(1)	Left thalamus, caudate	Anhedonia (31)
	(2)	Right thalamus, putamen	
	(3)	Left inferior frontal gyrus	
	(4)	Right inferior frontal gyrus	
	(5)	Pons	
Dorsal default mode network (DDMN)	(1)	Medial prefrontal cortex, anterior cingulate cortex, orbitofrontal cortex	Anhedonia (33) Rumination (34)
	(2)	Left angular gyrus	
	(3)	Right superior frontal gyrus	
	(4)	Posterior cingulate cortex, precuneus	
	(5)	Midcingulate cortex	
	(6)	Right angular gyrus	
	(7)	Left and right thalamus	
	(8)	Left hippocampus	
	(9)	Right hippocampus	
Left executive control network (LECN)	(1)	Left middle frontal gyrus, superior frontal gyrus	Rumination (32) Impaired cognitive reappraisal (14, 35)
	(2)	Left inferior frontal gyrus, orbitofrontal gyrus	
	(3)	Left superior parietal gyrus, inferior parietal gyrus, precuneus, angular gyrus	
	(4)	Left inferior temporal gyrus, middle temporal gyrus	
	(5)	Left thalamus	

*Three primary functional networks referenced throughout this main paper. For full list of networks, see Supplementary Table 1*.

### 2.4. Pairwise Maximum Entropy Model

**Figure 1:**
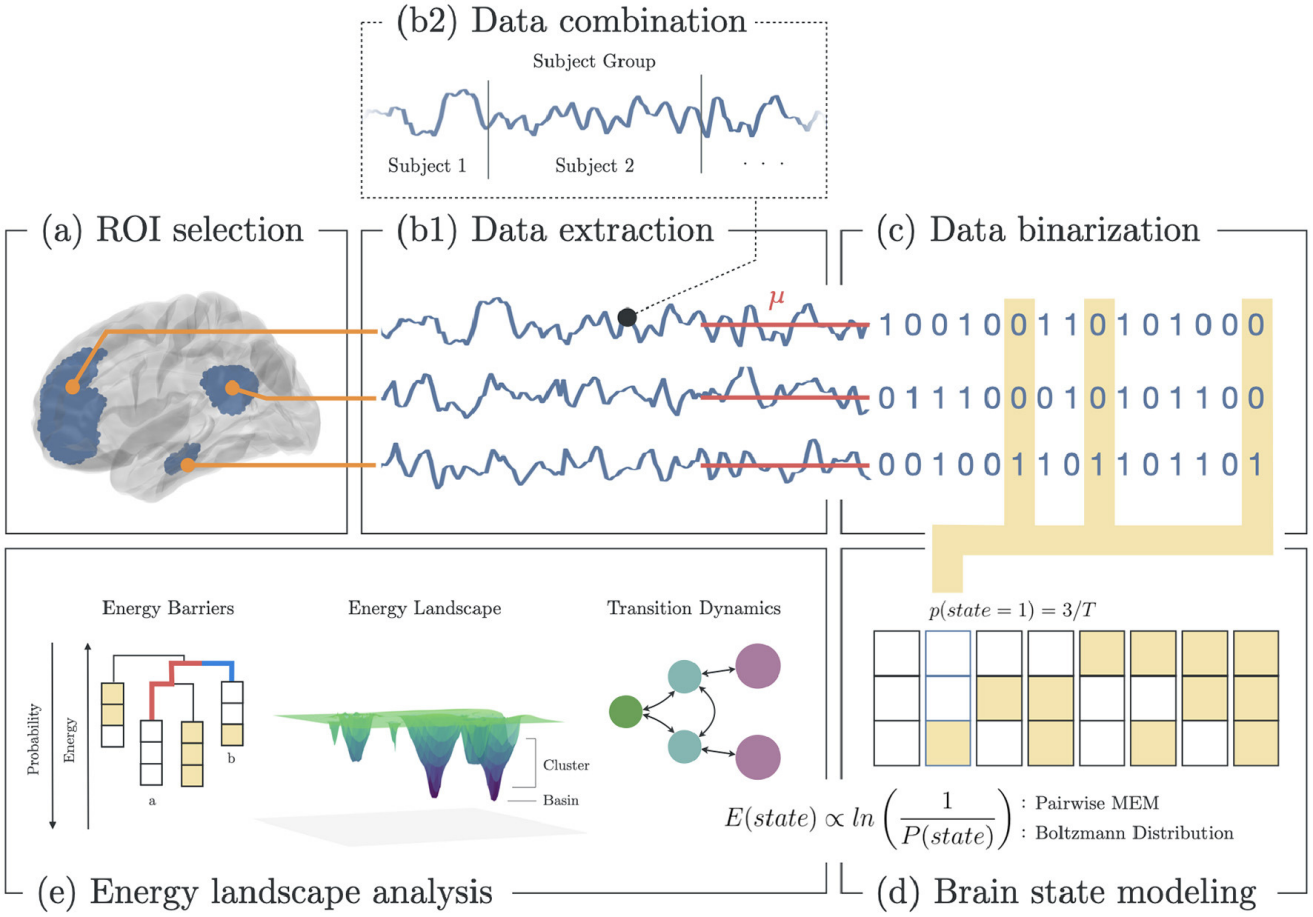
Methodology for energy landscape analysis. **(a)** Selection of ROI based on functional brain networks associated with depression. **(b1)** Extraction of fMRI BOLD signals from each ROI. **(b2)** All signals from subjects within group (healthy, melancholic, non-melancholic) are combined. **(c)** Binarization of each signal using the mean BOLD value for each ROI. **(d)** Estimation of the state energy based on the emprical state frequency/probability. **(e)** Energy landscape analysis, which includes analyses of state energy levels, basin state landscape, and state transition dynamics. Note that as the energy of a state increases, its probability of occurrence decreases.

To compare the brain dynamics of melancholic and non-melancholic participants, we implemented the Energy Landscape Analysis (ELA) method which utilizes the Pairwise Maximum Entropy Model (P-MEM) (Figure 1) (36). In this study, ELA was conducted separately for each of the 12 distinct functional networks (see Supplementary Table 1).

In ELA, we began by combining the rs-fMRI data of all participants in a group (healthy, non-melancholic, melancholic) to create group-level dynamics model. In each group, the concatenated time series data were extracted from the corresponding ROIs of the chosen functional network (Figures 1A,B). Then, the time series data were converted to binarized signals, using the average signal value as the threshold (Figure 1C). At any time point, binarized signals were either 1 (active) or 0 (inactive). The threshold value was computed for each ROI across the combined data of the group participants.

Binarizing the signals allowed us to encode the time signals into brain state sequences, wherein a brain state was defined by the activity pattern, i.e., active and inactive ROIs, at a given time (Figures 1C,D). Given brain network with *n* ROIs, there are 2^*n*^ possible states. For each brain state σ, we computed the empirical probability *p*(σ),


(1) $$\begin{array}{l} p\left(\sigma \right)=\frac{n_{\sigma }}{T} \end{array}$$

where *n*_σ_ is the number of occurrences that the state σ appeared in the time series, and *T* is the total length of the time series (Figure 1D).

From the brain state probabilities, we defined the state energy using Boltzmann distribution (37),


(2) $$\begin{array}{l} P\left(\sigma |h,J\right)=\frac{\exp\left[-E\left(\sigma |h,J\right)\right]}{\sum_{\sigma^{\prime }}\left[-E\left(\sigma^{\prime }|h,J\right)\right]} \end{array}$$

where the sum in denominator is taken over all 2^*n*^ possible states (each state is denoted by σ′). In P-MEM, the state energy *E*(σ) in Equation 2 was restricted in the quadratic form, and expressed as:


(3) $$\begin{array}{l} E\left(\sigma |h,J\right)=-\overset{n}{\underset{i=1}{\sum }}h_{i}\sigma_{i}-\frac{1}{2}\overset{n}{\underset{i=1}{\sum }}\overset{n}{\underset{\begin{array}{c} j=1\\ j\neq i \end{array}}{\sum }}J_{i,j}\sigma_{i}\sigma_{j} \end{array}$$

with $h={\left[h_{i}\right]}_{i=1}^{n}$ and $J={\left[J_{i,j}\right]}_{i,j=1}^{n}$ corresponding to the individual ROI activity and the pairwise ROI interaction, respectively. As implied by these equations, more active ROIs correspond to higher *h* and *J*, which leads to lower (more negative) energy (Equation 3), and higher occurrence probability (Equation 2) (36).

We then built the P-MEM from the model parameters *h* & *J* by maximizing their likelihood, as defined by


(4) $$\begin{array}{ll} \left(h,J\right) & =\underset{h,J}{arg max}L\left(h,J\right) \end{array}$$

(5) $$\begin{array}{ll} L\left(h,J\right) & =\overset{T}{\underset{t=1}{\prod }}P\left(\sigma \left(t\right)|h,J\right) \end{array}$$

for the entire time series of length *T*.

### 2.5. Energy Landscapes Analysis

With the P-MEMs for healthy, non-melancholic and melancholic groups, we analyzed the energy landscapes and transition dynamics of heterogeneous depressed brain networks (Figure 1E). The ELA features analyzed in this study are summarized in Supplementary Table 2.

To start with, basin state dendrograms were constructed by finding the basin states and their clusters. Basin states (or basins) are states with the lowest energy relative to their neighboring states. States are said to be neighbors if they differ in only one active/inactive region. If neighboring states have lower energy barriers relative to other states, they become part of the closest basin cluster (38). Basins are the core of energy landscapes since these states are presumed to be the most stable. Djikstra's algorithm was performed to search for the basins, and construct the leaves (basins) and branches (energy barriers) of the dendrogram. In Figure 1E: *Energy Barriers*, the energy barrier between states *a* and *b* are highlighted (red: *a* → *b*; blue: *b* → *a*). Here, *a* → *b* has higher barrier, and thus has lower probability of occurring (38).

Energy landscapes were then constructed to depict the energy level and cluster size of the basins. In Figure 1E: *Energy Landscape Analysis*, a 3D schematic diagram depicts the basins in an arbitrary state space. Here, basin clusters are plotted as concentric circles, where the basin is at the center, and neighboring states are on circles with radius equivalent to the their distance to the basin (i.e., a state that differs in one region with the basin has distance of 1, state the differs in two regions has distance of 2, etc.). Thus, each circle represented the states that were equidistant to the basin. Then for each circle (including the center), its depth was equivalent to the energy of the state with lowest energy (Equation 3) in that circle.

Since energy landscapes modeled only the group-level brain dynamics, we opted to analyze the brain dynamics of individual participants by computing the occurrence frequency of basins *f*(*A*) on individual fMRI time series. The occurrence frequencies of all basins (major and minor) are then summed for each group and for each network.

We also analyzed the transition dynamics on the energy landscapes. For each network, we selected two major basins as the basins with the lowest energy, *A*_1_ and *A*_2_; with *P*_1_ as clustered states for *A*_1_, *P*_2_ for *A*_2_. Then we computed the following participant-level transition rates,


(6) $$\begin{array}{ll} TR\left(A\to A^{\prime }\right) & =\frac{n\left(A_{1}\to A_{2}\right)+n\left(A_{2}\to A_{1}\right)}{T} \end{array}$$

(7) $$\begin{array}{ll} TR\left(P\to P^{\prime }\right) & =\frac{n\left(P_{1}\to P_{2}\right)+n\left(P_{2}\to P_{1}\right)}{T} \end{array}$$

where *n*(*U* → *V*) is the number of times the participant's time series entered state *U* and arrived at state *V*; *TR*(*A* → *A*′) is the major transition rate for major basins; *TR*(*P* → *P*′) is the peripheral transition rate for clusters of major basins. Moreover, we computed the staying rates of the states,


(8) $$\begin{array}{ll} SR\left(A\to A^{\prime }\right) & =\frac{n\left(A_{1}\to A_{1}\right)+n\left(A_{2}\to A_{2}\right)}{T} \end{array}$$

(9) $$\begin{array}{ll} SR\left(P\to P^{\prime }\right) & =\frac{n\left(P_{1}\to P_{1}\right)+n\left(P_{2}\to P_{2}\right)}{T} \end{array}$$

where this time we counted the number of times the participant's brain activity stayed on the major basins (*SR*(*A* → *A*′)), or the periphery (*SR*(*P* → *P*′)). The transition rates were used to define the *Traveling Score*, which measured the amount of times the brain successfully *traveled* from one major basin to another (Equation 10, **Figure 5A**). Similarly, the staying rates were used to define the *Lingering Score*, which measured the amount of times the brain *lingered* on a basin or along its periphery (Equation 11, **Figure 5A**),


(10) $$\begin{array}{ll} Traveling\;Score & =\frac{TR\left(A\to A^{\prime }\right)}{TR\left(P\to P^{\prime }\right)} \end{array}$$

(11) $$\begin{array}{ll} Linger\;Score & =SR\left(A\to A^{\prime }\right)+SR\left(P\to P^{\prime }\right) \end{array}$$

Note that the traveling score (Equation 10) is also known as efficiency score, which is the index of ease of transitions (38).

### 2.6. Statistical Group Differences

One-way Analysis of Variance (ANOVA) was performed to find significant group differences (healthy vs. non-melancholic vs. melancholic) in participants' age, IQ, BDI-II and SHAPS (Snaith-Hamilton Pleasure Scale) scores. Bonferroni correction was applied to compensate for multiple group comparisons. Similarly, Chi-squared test was performed for group differences in sex. Group differences are presumed to be statistically significant at *p* < 0.05 for both types of statistical analyses. One-way ANOVA with Bonferroni correction was also applied to test for statistical significance of results when comparing individual- and group-level differences in energy landscape characteristics such as basin frequency and transition rates. These results were also verified by Kruskal-Wallis test to cope with non-normally distributed data as well.

### 2.7. Depressive Symptoms Correlation

Finally, we investigated the correlation between depressive symptoms and basin characteristics. Two criteria were used for analyzing depressive symptoms: BDI-II for depression diagnosis (23), and SHAPS for assessing anhedonia (39). Multivariate linear regression model were fitted on the data, with *p* < 0.05 deemed as statistically significant relationship between the variables.

## 3. Results

### 3.1. Depressive Symptom Severity

We found significant differences between healthy, non-melancholic, and melancholic groups in terms of BDI-II and SHAPS score (Table 1). For both criteria, the melancholic group had higher average scores (*p* < 0.05, *Healthy* *vs*. *Melancholic*; *p* < 0.001, *Non*−*melancholic* *vs*. *Melancholic*), followed by non-melancholic group (*p* < 0.001, *H* *vs*. *N*). The results were consistent with many previous reports (40–42).

In terms of other demographical factors, we found no significant group differences in sex distribution, age, and IQ levels of the participants. However, there was significant difference between the number of melancholic and non-melancholic participants recruited on four different sites (Table 1).

### 3.2. Basins and Energy Barriers

**Figure 2:**
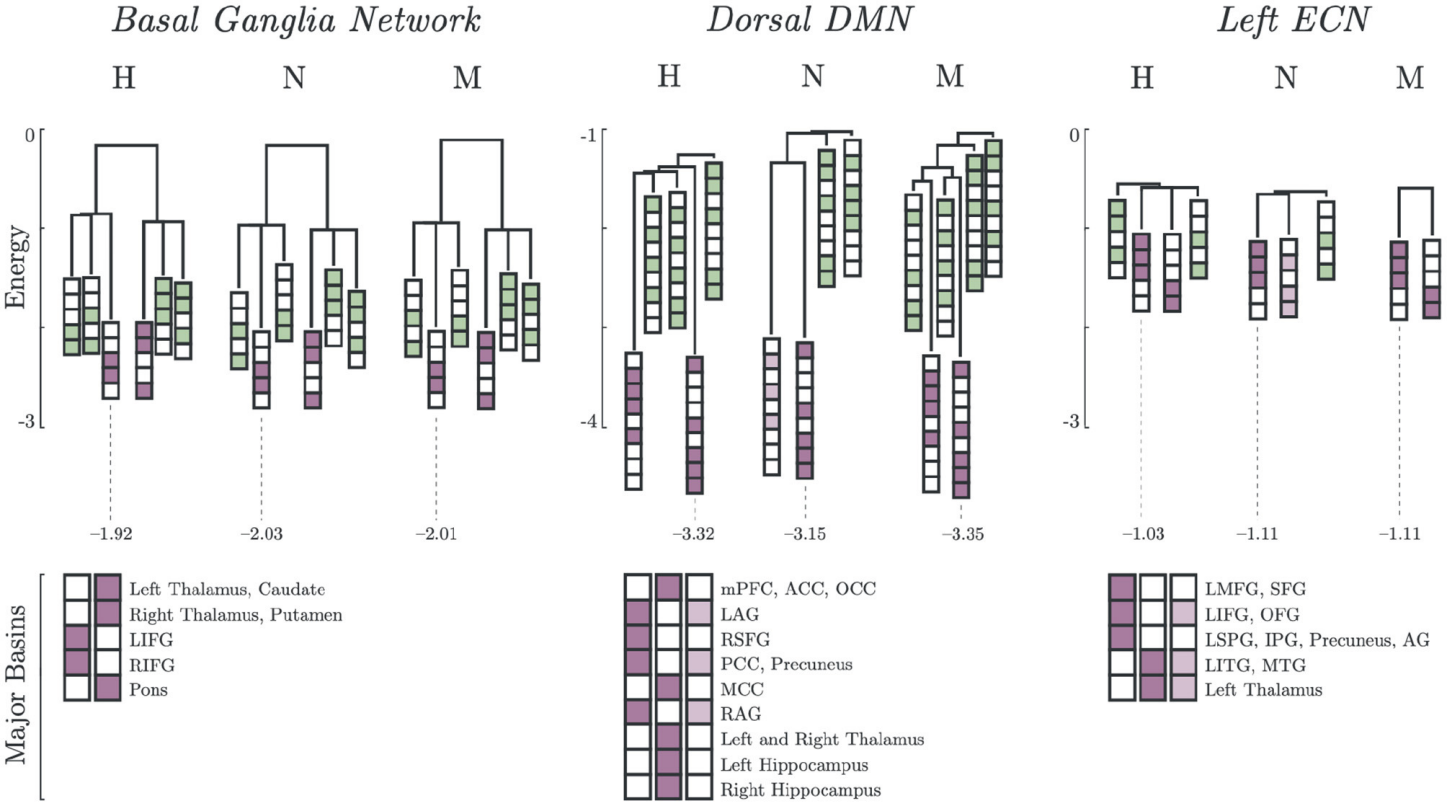
Major and minor basin states. Major basin states (purple) and minor basin states (green) for Healthy, Non-Melancholic, and Melancholic groups on BGN, DDMN, and LECN. For each state, colored boxes correspond to activated regions, while white boxes are inactive. Energies of the deepest major basins for each group are also indicated (see Supplementary Table 3 for summary of all major basin energies).

The basin dendrograms of each group for all network revealed differences in major basins and energy levels. The dendrograms for Basal Ganglia Network (BGN), Dorsal DMN (DDMN), and Left ECN (LECN) are shown in Figure 2. Here, the major basins (purple) were deeper (i.e., had relatively lower energy) than the minor basins (green).

In both BGN and LECN, the non-melancholic and melancholic groups had deeper major basins than the healthy group. Remarkably, a different pattern can be observed in the DDMN, where the non-melancholic group had shallower major basins than the healthy and melancholic groups.

Furthermore, some major basins were unique to specific groups. For example, non-melancholic group had a unique major basin in DDMN, where the left and right angular gyri (LAG, RAG), posterior cingulate cortex (PCC), and precuneus regions were activated. Similarly on LECN, the non-melancholic group also had a unique major basin, where left inferior frontal gyrus (LIFG), orbitofrontal gyrus (OFG), left inferior temporal gyrus (LITG), middle temporal gyrus (MTG), and left thalamus regions were activated. Complete results for basin energies are reported in Supplementary Table 3.

Lastly, the major basin pairs had antisynchronized activations (Figure 2). In BGN, the Left and Right Inferior Frontal Gyrus (LIFG, RIFG) were antisynchronized with the Left and Right Thalamus, Caudate, Putamen and Pons. In DDMN, the Left and Right Angular Gyrus (LAG, RAG), Right Superior Frontal Gyrus (RSFG), Posterior Cingulate Cortex (PCC) and Precuneus were synchronized with each other. A similar pattern can be observed in LECN, where the Left Inferior Temporal Gyrus (LITG), Middle Temporal Gyrus (MTG) and Left Thalamus were synchronized.

### 3.3. Basin Size and Energy Landscapes

**Figure 3:**
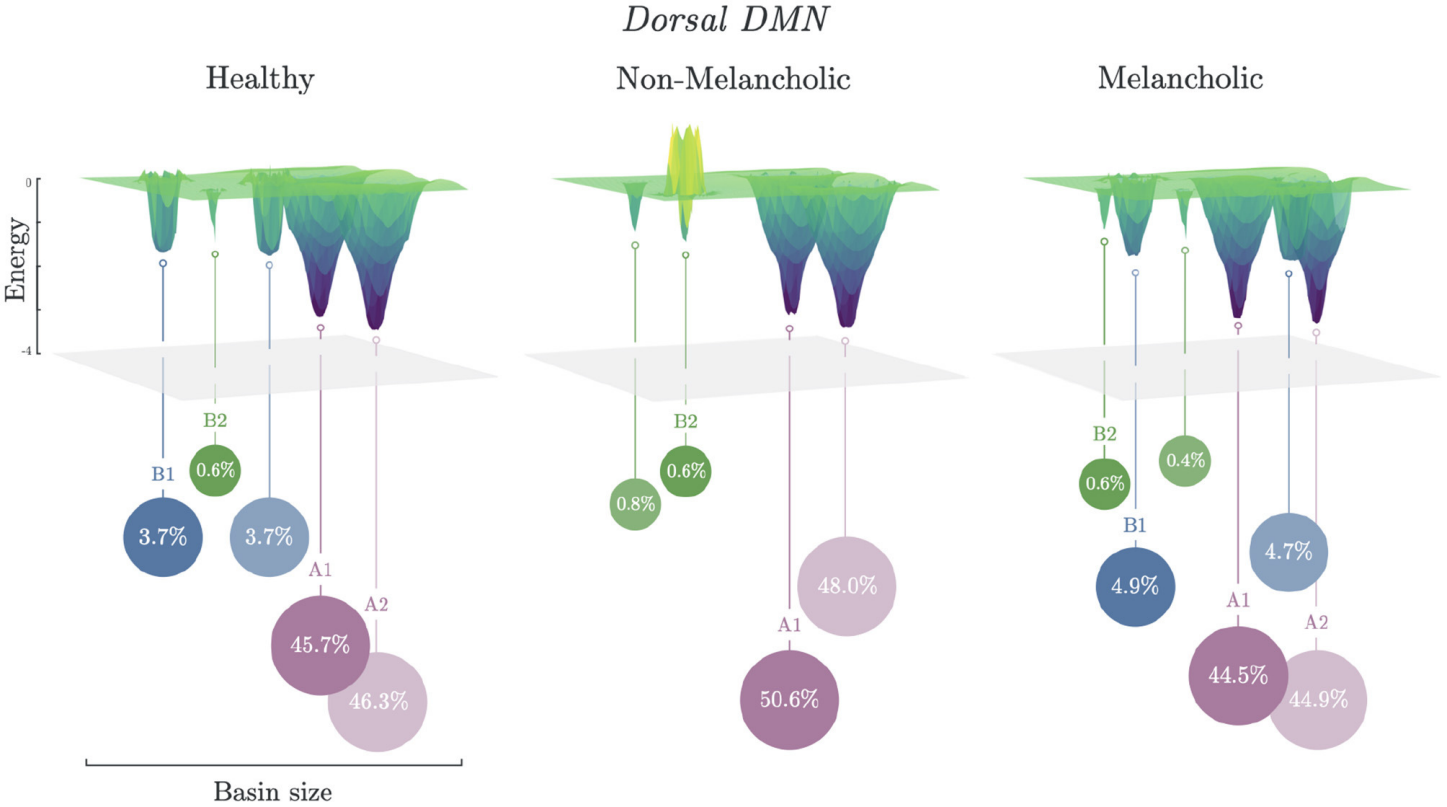
Energy landscapes of healthy, non-melancholic and melancholic groups. Schematic diagram of energy landscapes for Healthy, Non-melancholic, and Melancholic groups on DDMN. States are clustered to the their nearest basin state (Section 2.5). States A1 (*S*_279_) and A2 (*S*_232_) are the common major basins among the groups, while B1 (*S*_340_) and B2 (*S*_419_) are the common minor basins (Supplementary Table 3).

Energy landscapes are then constructed based on the basin information. In Figure 3, different energy landscapes can be observed from each group in DDMN. The melancholic group has the most number of basins (*n* = 6), followed by healthy group (*n* = 5), then non-melancholic group (*n* = 4). Because of this shortage of basins, the non-melancholic has relatively larger major basins (*s*_*A*_ = 98.6%), compared to healthy (*s*_*A*_ = 92.0%) and melancholic groups (*s*_*A*_ = 89.4%). Note that, as mentioned previously, the non-melancholic group has a unique major basin (*A*_2_). Also, there is a *hill* on basin cluster *B*_2_ of the non-melancholic group, which may be inferred as highly unstable cluster of states. This *hill* state cluster is absent in the other groups. We compared the basin sizes (number of states clustered to the basin) of each group for each network but found only minimal differences (e.g., *p* = 0.08, *N vs. M, DDMN*). Complete results for basin sizes are reported in Supplementary Figure 1 and Supplementary Table 3.

### 3.4. Basin Frequency

**Figure 4:**
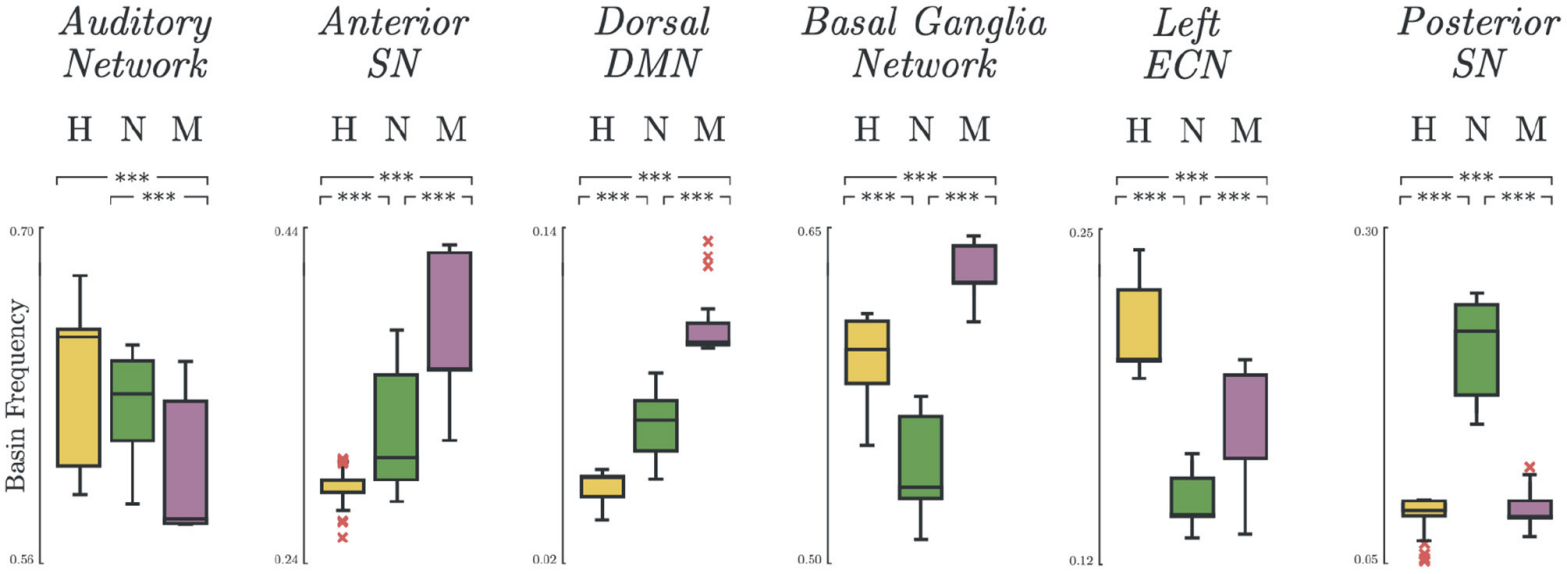
Basin states frequency. Occurrence frequency of basins for Healthy, Non-melancholic, and Melancholic groups on various brain networks. (***) *p* < 0.005; between-group, pairwise comparison of basin frequencies using one-way ANOVA with Bonferroni correction.

Significant group differences were found on the basin frequencies across all selected networks. The most notable results are summarized in Figure 4. In some networks, there was a natural decreasing trend from healthy to melancholic group (e.g., *p* < 0.005; *H vs. M, N vs. M*; *Auditory Network*). In other networks, an opposite, increasing trend appeared (e.g., *p* < 0.005; *H vs. N, H vs. M, N vs. M*; *Anterior Salience Network, DDMN*). And for the rest, the non-melancholic group had either the lowest (e.g., *p* < 0.005; *H vs. N, H vs. M, N vs. M*; *BGN, LECN*) or highest frequency (e.g., *p* < 0.005; *H vs. N, H vs. M, N vs. M*; *Posterior Salience Network*). Similar results for the other functional networks are summarized in Supplementary Table 4.

### 3.5. Brain State Transition Dynamics

**Figure 5:**
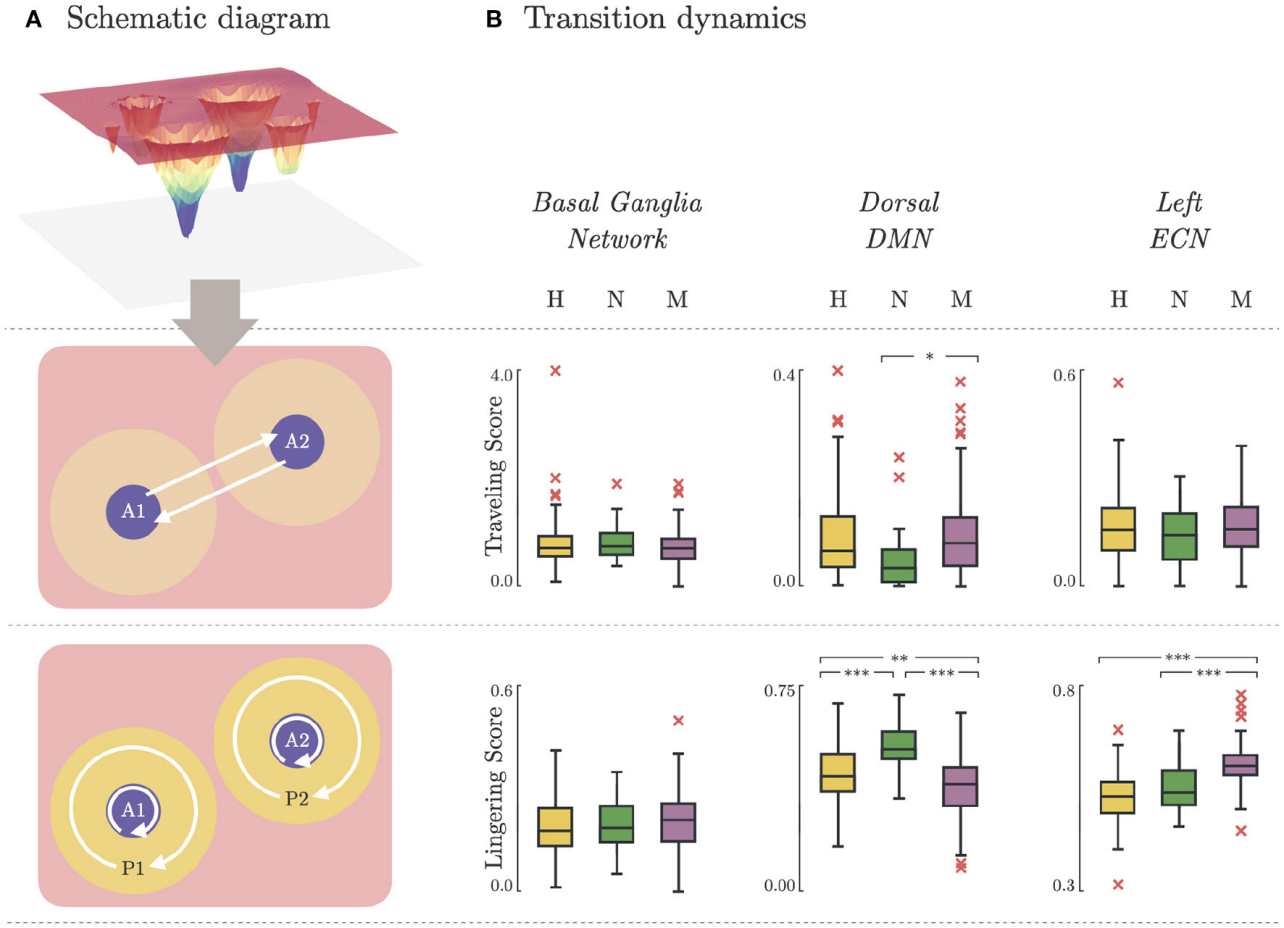
Brain state transition dynamics. **(A)** Schematic diagram illustrating the transition dynamics for traveling score (basin-to-basin; Equation 10) and lingering score (within-basin or within-peripheral; Equation 11). **(B)** Traveling and lingering scores for Healthy, Non-melancholic, and Melancholic groups on BGN, DDMN, and LECN. (*)*p* < 0.05, (**)*p* < 0.01, and (***)*p* < 0.005; between-group, pairwise comparison of traveling/lingering scores using one-way ANOVA with Bonferroni correction.

Depression heterogeneity was also evident when comparing the transition dynamics in some networks. As shown in Figure 5B, there were significant differences between non-melancholic and melancholic groups in traveling scores (*p* < 0.05; *N vs. M*; *DDMN*), and lingering scores (*p* < 0.01; *N vs. M*; *DDMN, LECN*) of individual participants. There was also significant group differences in the lingering scores between healthy and non-melancholic (*p* < 0.01; *H vs. N*; *DDMN*), and healthy and melancholic groups (*p* < 0.01; *H vs. M*; *DDMN, LECN*). Furthermore, there is significant increase in lingering scores of melancholic group in LECN (*Lingering* = 0.61 ± 0.06) as compared to non-melancholic (*Lingering* = 0.55 ± 0.06; *p* < 0.005) and healthy groups (*Lingering* = 0.53 ± 0.06; *p* < 0.005). Results for other functional networks are summarized in Supplementary Table 5.

## 4. Discussion

In this study, we characterized the brain dynamics of melancholic depression by analyzing the energy landscape models constructed from temporal rs-fMRI signals. The distinguishing energy landscape features of melancholic depression included major basin energy barriers, basin frequency, and state transition dynamics. Statistical results were consistent across our analyses on 12 functional brain networks, which indicates the robustness and discriminative power of our model. Moreover, these results agree with existing studies on melancholic features, such as depression severity (33, 40, 43), anhedonia (33, 44), and rumination (45, 46).

### 4.1. Confirmation of Depression Heterogeneity

Our first goal was to show heterogeneity in our depressed participants. Depression heterogeneity has plagued research advancement on the diagnosis and treatment of the disorder (47–49). Considerable efforts has been made toward drawing boundaries within the currently accepted definition of major depressive disorder (47, 48), suggesting that investigations on individual depressive symptoms and their interactions may lead to new discoveries (49).

Melancholia is recognized as a specifier for MDD, characterized by symptoms that overlap with MDD such as anhedonia, excessive guilt, psychomotor disturbance, cognitive impairment, weight loss, and worse mood in the morning (2, 50). Although melancholic depression is not anymore considered as a depression subtype by DSM-5 (which instead uses the technical term *depression with melancholic features*) (50, 51), its clinical characteristics distinguishes it from non-melancholic and atypical depression (43). Our analysis results confirm such distinctions, as observed from significant differences between melancholic and non-melancholic groups in BDI-II and anhedonia scores (Table 1); basin frequencies on 12 networks (Supplementary Table 4); and transition dynamics scores in DDMN, LECN, PSN, RECN, VDMN, and VSPN (Supplementary Table 5).

### 4.2. Melancholia and Depression Severity

Next, we sought meaningful relationship between energy landscape signatures and depression severity. Melancholic depression is associated with greater symptom severity in comparison to depression with non-melancholic features (40, 43). This association was evident in our participant-level analysis of BDI-II scores, where the melancholic group scored significantly higher than the other groups (Table 1).

Readers may wonder if energy landscape features can be a proxy to evaluate some symptoms associated with melancholic features. To check this possibility, we performed regression analysis on basin frequencies and depression severity score (BDI-II). But despite having clear boundaries among the groups, no significant correlation were found in any of the networks (Supplementary Figure 2A).

The order of increasing severity from healthy, to non-melancholic, to melancholic depression may seem intuitive. However, some studies argue against the severity-based categorization of melancholic depression (52, 53), and do not identify melancholia as a “more severe” form of depression (52). This is evident in Supplementary Figure 2A, where the BDI-II scores for non-melancholic and melancholic groups coincide. In fact, we can even argue that the basin frequency of our energy landscape model were better than the depressive scores in discriminating melancholic depression (Figure 4).

### 4.3. Melancholia and Anhedonia

Following depression severity, we then tested if the correlation with basin frequency would also hold true for anhedonia. Anhedonia is one of the overlapping symptoms for both melancholia and depression (2, 42). This effect was observed from the statistical analysis of the subject demographics, where melancholic group has significantly higher SHAPS score for anhedonia (Table 1).

Similar to BDI-II scores, there were no significant correlation between SHAPS scores and basin frequencies (Supplementary Figure 2B). However, a closer inspection of results in the DDMN reveals a plausible connection between these features. Studies report that the decrease in functional connectivity of dorsomedial prefrontal cortex (*dmPFC*) with posterior cingulate cortex / precuneus (*PCC/PCUN*) is related to depression severity and anhedonia (33, 44).

Looking at the major basins found by our model in DDMN (Figure 2), the major basin pairs for all groups have antisynchronized activations of mPFC and PCUN (*S*_279_:{*mPFC* = 1, *PCC*/*PCUN* = 0}, H, N, M; *S*_232_:{*mPFC* = 0, *PCC*/*PCUN* = 1}, H, M; *S*_168_:{*mPFC* = 1, *PCC*/*PCUN* = 0}, N). This decoupling between mPFC and PCC/PCUN may be attributed to impaired reward anticipation, which is a key feature of anhedonia (44). Although current research has not yet established the direct connection between dynamic functional connectivity and energy landscape model (38), our results provide insight to the potential connection between these models.

### 4.4. Melancholia and Ruminative State

Lastly, we investigated the possible presence of ruminative states in the energy landscapes. Rumination is the tendency to dwell on the same, usually negative, thoughts for prolonged periods of time. Currently, rumination is not recognized a diagnostic feature for melancholic depression. However, since past studies have noted the significant correlation of rumination to both melancholy (45) and depression (54), we explored the energy landscape characteristics to provide more evidences on this correlations. Ruminative thinking is marked by increased connectivity in DMN (55), and decreased activity in left dorsolateral prefrontal cortex (*left dlPFC*; equivalent to *LECN*) (46). In terms of our energy landscape model, we define dynamic activity as being able to transition from one basin to another. This is in contrast to static activity, which tends to stay at the same basin. We derived two measures for brain activity: *traveling score* (Equation 10) for dynamic activity, and *lingering score* (Equation 11) for static activity. Thus, high traveling score and low lingering score would imply a more active network.

The significant increase of lingering scores of the melancholic group in LECN (Figure 5) could imply that the melancholic group has greater tendency to be “stuck” in a basin, or within its cluster. This may be related to the decreased activity in left dlPFC found in rumination (46). On the contrary, in the dorsal part of DMN (*DDMN*), the lingering scores of melancholic group (*Lingering* = 0.55 ± 0.07) are significantly lower than healthy (*Lingering* = 0.58 ± 0.06; *p* < 0.01) and non-melancholic groups (*Lingering* = 0.63 ± 0.05; *p* < 0.005). Although this may support previous studies showing increased connectivity in DMN during ruminative thinking, analyzing the lingering scores in ventral part of DMN (*VDMN*) leads to contradicting results (55). Nevertheless, the traveling scores in DDMN are significantly higher in melancholic group (*Traveling* = 0.10 ± 0.09) than in non-melancholic group (*Traveling* = 0.05 ± 0.06; *p* < 0.05). For LECN and VDMN, there were no significant differences in traveling scores between groups.

### 4.5. Limitations and Future Directions

Unlike typical functional connectivity (FC) models that are based on correlation between two regions (i.e., using Pearson's correlation coefficient), the ELA model does not assume that pairwise interactions of regions are independent from each other (17). This allows our model to more accurately capture the global activity patterns that may possibly be overlooked by FC-based models (17). Some FC-based models try to work around this limitation by using a different FC metric (such as precision matrix), or by introducing a sliding window in the analysis (also known as dynamic FCA) (56).

Hidden Markov Model (HMM) is another common brain dynamics model for resting-state fMRI. In comparison to ELA which utilizes pairwise MEM, HMM is more complex, and may be more expressive (57). Thus, in the future, our ELA results can serve as a baseline for deeper analysis on brain dynamics of melancholic depression using models such as HMM.

Furthermore, our present study have other limitations. ELA assumes a memoryless process, such that predicting the next state only depends on the current state, and not the past states (38). This saves us memory space and processing time (58), but this only works on systems at equilibrium, which typically requires huge amount of data (59). To address this problem, first, we decided to use resting-state fMRI data since these are expected as equilibrium states (60). Then, we combined the fMRI time signals of all participants in a group, to essentially produce “long” temporal data. Since there are substantially more healthy participants, it is possible that the model for the healthy group has converged closer to equilibrium than the two depressed subgroups.

Although concatenation of individual fMRI data should only have minimal effect to our analysis due to the memorylessness aspect of ELA (61), it should be noted that combining participants fMRI data could still potentially introduce bias due to BOLD signal differences across participants. We addressed this by using individual subjects' mean fMRI BOLD signals as threshold for binarizing the signals. However, it is recommended to further investigate the implications of data concatenation, and the ways to mitigate the intensity differences in individual fMRI.

Our selection of ROIs relies on the parcellation method [Shirer Atlas, (28)] and availability of data. As such, we removed some ROIs due to unreliable or insufficient data. Inclusion of these ROIs might affect the results. Choosing a different parcellation method might also produce different results. Thus, testing the robustness and consistency of our model on complete sets of ROIs or with different parcellation methods would be a reasonable step in the future.

It is often pointed out that the site difference could be a potential confounding factor for the observed BOLD signals (62). In fact, when we applied two-way ANOVA considering two factors of participant groups and site ID to basin frequencies, all main effects and their interaction were statistically significant (Supplementary Table 4). While this still supports that basin frequencies across different networks could differentiate participants suffering from melancholic and non-melancholic MDD in a population level, its reliability as a biomarker for the personalized medicine should never be overestimated. Thus, the potential site bias is a limitation in the present study and we should address the issue in our future work.

Even though we hypothesized *a priori* that the energy landscape of melancholic depression will be different from non-melancholic due to depression heterogeneity, our analyses and interpretations of the results were done *post-hoc*. For statistical analyses, we applied Bonferroni correction to compensate for multiple comparison tests that would increase the risk of Type 1 error (63).

Our discussion on the relation between melancholia and rumination is more suggestive than conclusive since we lack rumination quantifiers to analyze with our models. In the future, it would be crucial to record ruminative tendencies of participants using standardized measures such as Ruminative Response Scale (RRS) (64).

Finally, we would like to emphasize that melancholia, in itself, is also heterogeneous (50). It is characterized by multiple symptoms, and thus its severity and symptoms may vary from one person to another. Current biomarker models focus on the distinguishing features of melancholic depression on a group level (9, 10, 14). ELA allows us to study not only the group-wide brain dynamics of melancholic depression, but also the individual nuances in brain states and functional region interactions. In the future, it would be beneficial to study more of these individual differences so we can directly confront the issue of heterogeneity.

### 4.6. Conclusion

Melancholic depression is a debilitating disorder that robs a person the pleasure and excitement from activities they used to enjoy. In this study, we developed an energy landscape model to better understand the brain dynamics involved in melancholic depression. Relative to healthy and non-melancholic groups, the melancholic group showed significant differences on basin energy, basin frequency, and transition dynamics in several functional networks. Moreover, possible connections were traced between major basins and depressive symptom scores such as depression severity and anhedonia. Taken together, these results suggest that melancholic depression is a distinct disorder, and thus should be diagnosed and treated with utmost precision and care.

## Data Availability Statement

The raw data supporting the conclusions of this article will be made available by the authors, without undue reservation.

## Ethics Statement

The studies involving human participants were reviewed and approved by the Ethics Committee of Hiroshima University. The patients/participants provided their written informed consent to participate in this study. Written informed consent was not obtained from the individual(s) for the publication of any potentially identifiable images or data included in this article.

## Author Contributions

PR: methodology, software, investigation, writing—original draft, and writing—review and editing. MT: resources and writing—review and editing. TN: conceptualization, supervision, and writing—review and editing. NI, AF, and GO: resources. YO and SY: supervision and funding acquisition. KI: supervision and writing—review and editing. JY: conceptualization, methodology, investigation, writing—original draft, writing—review and editing, and funding. All authors contributed to the article and approved the submitted version.

## Funding

This research was supported by the Strategic Research Program for Brain Sciences (Integrated Research on Depression, Dementia and Development Disorders) from the Japan Agency for Medical Research and Development, AMED, grant nos. JP20dm0107093 and 20dm0107096. This work is also partially supported by JST-Mirai Program grant no. JPMJMI20D6, Japan; and JSPS KAKENHI grant nos. JP21H00949, JP21K07521, JP20H00625, and JP20K06874.

## Conflict of Interest

The authors declare that the research was conducted in the absence of any commercial or financial relationships that could be construed as a potential conflict of interest.

## Publisher's Note

All claims expressed in this article are solely those of the authors and do not necessarily represent those of their affiliated organizations, or those of the publisher, the editors and the reviewers. Any product that may be evaluated in this article, or claim that may be made by its manufacturer, is not guaranteed or endorsed by the publisher.
