# Supplementary material for: Modeling Heterogeneous Brain Dynamics of Depression and Melancholia Using Energy Landscape Analysis
Source: Front Psychiatry. 2021 Nov 25;12:780997. doi: 10.3389/fpsyt.2021.780997 (PMC8656401; doi:10.3389/fpsyt.2021.780997)
Supplement: Supplementary file 1 [file Data_Sheet_1.pdf]

Supplementary Material

1 FUNCTIONAL BRAIN NETWORKS

Complete list of functional networks is provided in Table S1. In this table, the anatomical regions are listed in order for each ROI. This ordering is important for defining the brain states, such that given ROIs $\begin{bmatrix} R_1 & R_2 & R_3 \end{bmatrix}$, the state S_1 corresponds to activated regions $\begin{bmatrix} 0 & 0 & 1 \end{bmatrix}$. In other words, the state index is equivalent to the binary representation of the activated regions in the state ($index_{10} = activation_2$).

For each network, the associated depressive symptoms, such as anhedonia, rumination, and negative feelings, are cross-referenced to existing depression studies.

Table S1. Complete list of functional brain networks associated with depression

Network		Anatomical Locations of Functional ROIs
Anterior Salience Network (ASN)	(1)	Left Middle Frontal Gyrus
	(2)	Left Insula
	(3)	Anterior Cingulate Cortex, Medial Prefrontal Cortex, Supplementary Motor Area
	(4)	Right Middle Frontal Gyrus
	(5)	Right Insula
Auditory Network (AN)	(1)	Left Superior Temporal Gyrus, Heschl's Gyrus
	(2)	Right Superior Temporal Gyrus
	(3)	Right Thalamus
Basal Ganglia Network (BGN)	(1)	Left Thalamus, Caudate
	(2)	Right Thalamus, Putamen
	(3)	Left Inferior Frontal Gyrus
	(4)	Right Inferior Frontal Gyrus
	(5)	Pons

Table S1. Complete list of functional brain networks associated with depression (continued)

Network		Anatomical Locations of Functional ROIs
Dorsal Default Mode Network (DDMN)	(1)	Medial Prefrontal Cortex, Anterior Cingulate Cortex, Orbitofrontal Cortex
	(2)	Left Angular Gyrus
	(3)	Right Superior Frontal Gyrus
	(4)	Posterior Cingulate Cortex, Precuneus
	(5)	Midcingulate Cortex
	(6)	Right Angular Gyrus
	(7)	Left and Right Thalamus
	(8)	Left Hippocampus
	(9)	Right Hippocampus
Language Network (LN)	(1)	Inferior Frontal Gyrus
	(2)	Left Middle Temporal Gyrus
	(3)	Left Middle Temporal Gyrus, Angular Gyrus
	(4)	Left Middle Temporal Gyrus, Superior Temporal Gyrus, Supramarginal Gyrus, Angular Gyrus
	(5)	Right Inferior Frontal Gyrus
	(6)	Right Supramarginal Gyrus, Superior Temporal Gyrus, Middle Temporal Gyrus
Left Executive Control Network (LECN)	(1)	Left Middle Frontal Gyrus, Superior Frontal Gyrus
	(2)	Left Inferior Frontal Gyrus, Orbitofrontal Gyrus
	(3)	Left Superior Parietal Gyrus, Inferior Parietal Gyrus, Precuneus, Angular Gyrus
	(4)	Left Inferior Temporal Gyrus, Middle Temporal Gyrus
	(5)	Left Thalamus

Table S1. Complete list of functional brain networks associated with depression (continued)

Network		Anatomical Locations of Functional ROIs
Posterior Salience Network (PSN)	(1)	Left Middle Frontal Gyrus
	(2)	Left Supramarginal Gyrus, Inferior Parietal Gyrus
	(3)	Left Precuneus
	(4)	Right Midcingulate Cortex
	(5)	Right Superior Parietal Gyrus, Precuneus
	(6)	Right Supramarginal Gyrus, Inferior Parietal Gyrus
	(7)	Left Thalamus
	(8)	Left Posterior Insula, Putamen
	(9)	Right Thalamus
	(10)	Right Posterior Insula
Precuneus Network (PN)	(1)	Midcingulate Cortex, Posterior Cingulate Cortex
	(2)	Precuneus
	(3)	Left Angular Gyrus
	(4)	Right Angular Gyrus
Right Executive Control Network (RECN)	(1)	Right Middle Frontal Gyrus, Right Superior Frontal Gyrus
	(2)	Right Middle Frontal Gyrus
	(3)	Right Inferior Parietal Gyrus, Supramarginal Gyrus, Angular Gyrus
	(4)	Right Superior Frontal Gyrus
	(5)	Right Caudate

Table S1. Complete list of functional brain networks associated with depression (continued)

Network		Anatomical Locations of Functional ROIs
Sensorimotor Network (SMN)	(1)	Left Precentral Gyrus, Postcentral Gyrus
	(2)	Right Precentral Gyrus, Postcentral Gyrus
	(3)	Right Supplementary Motor Area
	(4)	Left Thalamus
	(5)	Right Thalamus
Ventral Default Mode Network (VDMN)	(1)	Left Retrosplenial Cortex, Posterior Cingulate Cortex
	(2)	Left Middle Frontal Gyrus
	(3)	Left Parahippocampal Gyrus
	(4)	Left Middle Occipital Gyrus
	(5)	Right Retrosplenial Cortex, Posterior Cingulate Cortex
	(6)	Precuneus
	(7)	Right Superior Frontal Gyrus, Middle Frontal Gyrus
	(8)	Right Parahippocampal Gyrus
	(9)	Right Angular Gyrus, Middle Occipital Gyrus
Visuospatial Network (VSPN)	(1)	Left Middle Frontal Gyrus, Superior Frontal Gyrus, Precentral Gyrus
	(2)	Left Inferior Parietal Sulcus
	(3)	Left Frontal Operculum, Inferior Frontal Gyrus
	(4)	Left Inferior Temporal Gyrus
	(5)	Right Middle Frontal Gyrus
	(6)	Right Inferior Parietal Lobule
	(7)	Right Frontal Operculum, Inferior Frontal Gyrus
	(8)	Right Middle Temporal Gyrus

2 ENERGY LANDSCAPE RESULTS

Complete results of energy landscape analyses are presented here. Analyses were conducted separately for each group (healthy, non-melancholic, and melancholic) and on each functional network (Table S1).

Table S2. Energy landscape features

Feature	Description	
Basin states	States with the lowest energy relative to neighbors	Figure 2, Figure 3, Figure S1
Major basin states	Two of the basin states with the lowest energy	Figure 2, Table S3
Minor basin states	Non-major basin states	Figure 2
Basin size	Number of neighboring states clustered to a basin state	Figure 3, Figure S1, Table S3
Basin state energy	Energy level of a basin state	Equation 3, Figure 2, Table S3
Basin state occurrence frequency	Number of times the basin state occurred in a subject's fMRI time series data	Figure 4, Table S4
Traveling score	Rate of successful transition from one major basin state to another	Figure 5, Table S5
Lingering score	Rate of staying within each of the major basin states, or their peripheral (clustered) states	Figure 5, Table S5

Table S3. Energy level and size of basins

	Healthy			Non-melancholic			Melancholic		
Network	B	E	Size	B	E	Size	B	E	Size
ASN	9	-1.78	43.75 %	9	-1.94	43.75 %	9	-1.91	43.75 %
	22	-1.74	43.75 %	22	-1.90	43.75 %	22	-1.91	43.75 %
	3	-0.85	6.25 %	3	-0.79	6.25 %	3	-0.94	6.25 %
	28	-0.83	6.25 %	28	-0.74	6.25 %	28	-0.92	6.25 %
AN	1	-3.00	50.00 %	1	-3.03	50.00 %	1	-3.02	50.00 %
	6	-2.97	50.00 %	6	-2.99	50.00 %	6	-2.99	50.00 %
BGN	6	-1.92	25.00 %	6	-2.03	28.12 %	6	-2.01	28.12 %
	25	-1.92	25.00 %	25	-2.00	28.12 %	25	-2.01	28.12 %
	26	-1.51	15.62 %	26	-1.60	12.50 %	26	-1.51	12.50 %
	28	-1.48	9.38 %	5	-1.60	12.50 %	5	-1.49	12.50 %
	3	-1.47	15.62 %	28	-1.38	9.38 %	28	-1.43	9.38 %
	5	-1.47	9.38 %	3	-1.34	9.38 %	3	-1.39	9.38 %
DDMN	279	-3.32	45.70 %	279	-3.15	50.59 %	279	-3.35	44.53 %
	232	-3.29	46.29 %	168	-3.13	48.05 %	232	-3.30	44.92 %
	340	-1.68	3.71 %	419	-1.20	0.59 %	340	-1.70	4.88 %
	171	-1.63	3.71 %	92	-1.08	0.78 %	139	-1.66	4.69 %
	419	-1.29	0.59 %				419	-1.25	0.59 %
							404	-1.13	0.39 %
LN	29	-1.51	37.50 %	34	-1.71	46.88 %	29	-1.46	43.75 %
	34	-1.51	37.50 %	29	-1.65	46.88 %	34	-1.45	43.75 %
	56	-1.19	10.94 %	56	-1.19	1.56 %	56	-1.14	6.25 %
	7	-1.19	10.94 %	7	-1.18	1.56 %	7	-1.11	6.25 %
	19	-0.95	1.56 %	19	-1.08	1.56 %			
	44	-0.93	1.56 %	44	-1.08	1.56 %			

Basin states, their energy and cluster size. See Section S1 for details on state indexing.
Major basins are listed on first two rows (highlighted in purple) for each group and network. Unique basins are shaded in light purple. For each group, basins are sorted from lowest energy, and then from largest size.

Table S3. Energy level and size of basins (continued)

	Healthy			Non-melancholic			Melancholic		
Network	B	E	Size	B	E	Size	B	E	Size
LECN	28	-1.03	46.88 %	28	-1.11	50.00 %	28	-1.11	50.00 %
	3	-1.03	46.88 %	11	-1.10	46.88 %	3	-1.09	50.00 %
	5	-0.68	3.12 %	5	-0.69	3.12 %			
	26	-0.68	3.12 %						
PSN	527	-3.00	18.16 %	789	-2.97	13.38 %	234	-2.93	16.50 %
	496	-2.95	17.09 %	234	-2.95	13.38 %	789	-2.90	17.19 %
	789	-2.88	10.55 %	79	-2.93	15.23 %	15	-2.64	20.70 %
	234	-2.85	11.72 %	944	-2.87	14.55 %	1008	-2.49	20.02 %
	79	-2.70	7.03 %	527	-2.81	8.01 %	794	-2.42	8.40 %
	229	-2.66	8.59 %	496	-2.77	7.91 %	229	-2.40	7.71 %
	794	-2.65	10.74 %	794	-2.68	11.13 %	287	-2.17	2.54 %
	944	-2.62	6.74 %	229	-2.68	10.94 %	736	-2.14	3.61 %
	287	-2.23	2.15 %	287	-2.35	1.46 %	378	-1.79	1.37 %
	736	-2.20	2.34 %	736	-2.32	1.37 %	906	-1.71	0.98 %
	341	-2.09	2.83 %	714	-1.98	1.17 %	117	-1.71	0.98 %
	117	-1.85	2.05 %	309	-1.95	1.46 %			
PN	12	-1.49	37.50 %	3	-1.44	37.50 %	12	-1.44	37.50 %
	3	-1.43	37.50 %	12	-1.42	37.50 %	3	-1.41	37.50 %
	10	-0.87	6.25 %	10	-0.86	6.25 %	10	-0.81	6.25 %
	5	-0.81	6.25 %	5	-0.70	6.25 %	5	-0.80	6.25 %
	6	-0.62	6.25 %	6	-0.62	6.25 %	9	-0.60	6.25 %
	9	-0.61	6.25 %	9	-0.60	6.25 %	6	-0.54	6.25 %
RECN	28	-1.05	31.25 %	28	-1.13	31.25 %	12	-1.12	37.50 %
	3	-1.03	28.12 %	3	-1.13	28.12 %	3	-1.11	37.50 %
	9	-0.99	12.50 %	10	-1.12	12.50 %	10	-0.90	6.25 %
	22	-0.99	12.50 %	21	-1.00	12.50 %	9	-0.86	6.25 %
	21	-0.96	9.38 %	9	-0.99	6.25 %	21	-0.86	6.25 %
	10	-0.96	6.25 %	22	-0.97	9.38 %	22	-0.84	6.25 %

Basin states, their energy and cluster size. See Section S1 for details on state indexing.
Major basins are listed on first two rows (highlighted in purple) for each group and network. Unique basins are shaded in light purple. For each group, basins are sorted from lowest energy, and then from largest size.

Table S3. Energy level and size of basins (continued)

	Healthy			Non-melancholic			Melancholic		
Network	B	E	Size	B	E	Size	B	E	Size
SMN	3	-2.12	50.00 %	3	-1.97	53.12 %	3	-2.00	50.00 %
	28	-2.09	50.00 %	28	-1.92	46.88 %	28	-1.96	50.00 %
VDMN	206	-2.82	20.31 %	206	-2.91	21.09 %	346	-3.03	25.20 %
	305	-2.75	19.73 %	305	-2.82	17.77 %	165	-3.03	24.02 %
	338	-2.72	21.29 %	338	-2.66	20.31 %	305	-2.71	19.73 %
	165	-2.72	21.68 %	165	-2.66	23.63 %	206	-2.70	21.29 %
	99	-1.85	8.59 %	99	-2.01	8.01 %	99	-1.69	5.08 %
	404	-1.83	8.40 %	412	-1.95	9.18 %	404	-1.67	4.69 %
VSPN	85	-2.80	21.09 %	170	-2.85	29.30 %	85	-2.79	26.56 %
	170	-2.76	21.09 %	85	-2.85	28.12 %	170	-2.74	23.83 %
	204	-2.20	20.70 %	51	-2.22	14.06 %	204	-2.10	17.19 %
	51	-2.19	20.70 %	204	-2.14	14.06 %	51	-2.09	16.80 %
	102	-1.75	6.25 %	102	-1.57	6.25 %	102	-1.88	6.25 %
	153	-1.74	6.25 %	153	-1.51	6.25 %	153	-1.86	6.25 %
	15	-1.26	1.95 %	240	-1.16	1.56 %	232	-1.76	2.73 %
	240	-1.24	1.95 %	15	-1.12	0.39 %	240	-1.13	0.39 %

Basin states, their energy and cluster size. See Section S1 for details on state indexing.
Major basins are listed on first two rows (highlighted in purple) for each group and network. Unique basins are shaded in light purple. For each group, basins are sorted from lowest energy, and then from largest size.

Table S4. Basin occurrence frequency

	Basin frequency			p-value					
Network	Healthy	Non-melancholic	Melancholic	(H,N)	(H,M)	(N,M)	KW[1]	Site[2]	G×S[3]
ASN	0.28 ± 0.01	0.31 ± 0.03	0.39 ± 0.04	***	***	***	***	***	***
AN	0.64 ± 0.03	0.62 ± 0.02	0.60 ± 0.03		***	***	***	***	***
BGN	0.59 ± 0.02	0.54 ± 0.02	0.63 ± 0.01	***	***	***	***	***	***
DDMN	0.05 ± 0.00	0.07 ± 0.01	0.11 ± 0.01	***	***	***	***	***	***
LN	0.23 ± 0.01	0.25 ± 0.01	0.20 ± 0.01	***	***	***	***	***	***
LECN	0.24 ± 0.02	0.15 ± 0.01	0.19 ± 0.03	***	***	***	***	***	***
PSN	0.09 ± 0.01	0.21 ± 0.04	0.10 ± 0.01	***	***	***	***	***	***
PN	0.69 ± 0.01	0.63 ± 0.01	0.65 ± 0.03	***	***	***	***	***	***
RECN	0.41 ± 0.01	0.34 ± 0.00	0.38 ± 0.01	***	***	***	***	***	***
SMN	0.44 ± 0.04	0.43 ± 0.02	0.30 ± 0.02		***	***	***	***	***
VDMN	0.06 ± 0.02	0.14 ± 0.01	0.09 ± 0.01	***	***	***	***	***	***
VSPN	0.13 ± 0.01	0.20 ± 0.01	0.20 ± 0.00	***	***	***	***	***	***

Occurrence frequency (mean ± std) of basins on individual participants' fMRI time signals.

(***) $p < 0.005$; between-group, pairwise comparison of basin frequencies using one-way ANOVA with Bonferroni correction

[1] Kruskal-Wallis test for non-normal distribution
[2] Two-way ANOVA test for significant differences across fMRI recording sites
[3] Two-way ANOVA test for significant interaction between group (H, N, M) and site (HUH, HRC, HKH, COI)

Table S5. Major basins transition dynamics

Network	Score	Healthy	Non-melancholic	Melancholic	p-value (H,N)	(H,M)	(N,M)	KW
ASN	Traveling	0.38 ± 0.23	0.41 ± 0.18	0.39 ± 0.18				
	Lingering	0.41 ± 0.07	0.38 ± 0.08	0.40 ± 0.08				
	TR(A)	0.08 ± 0.04	0.08 ± 0.04	0.08 ± 0.03				
	TR(P)	0.21 ± 0.04	0.21 ± 0.04	0.21 ± 0.04				
	SR(A)	0.09 ± 0.06	0.10 ± 0.06	0.10 ± 0.06				*
	SR(P)	0.32 ± 0.10	0.28 ± 0.11	0.30 ± 0.10				*
AN	Traveling	0.91 ± 0.37	0.85 ± 0.29	0.92 ± 0.41				
	Lingering	0.48 ± 0.06	0.47 ± 0.07	0.49 ± 0.06				
	TR(A)	0.15 ± 0.04	0.15 ± 0.04	0.15 ± 0.04				
	TR(P)	0.18 ± 0.04	0.19 ± 0.04	0.18 ± 0.04				
	SR(A)	0.31 ± 0.09	0.31 ± 0.09	0.31 ± 0.09				
	SR(P)	0.17 ± 0.07	0.16 ± 0.05	0.18 ± 0.08				
BGN	Traveling	0.78 ± 0.42	0.82 ± 0.35	0.75 ± 0.33				
	Lingering	0.19 ± 0.08	0.19 ± 0.08	0.21 ± 0.09				
	TR(A)	0.08 ± 0.03	0.09 ± 0.03	0.07 ± 0.03				
	TR(P)	0.11 ± 0.03	0.11 ± 0.03	0.11 ± 0.03				
	SR(A)	0.11 ± 0.07	0.12 ± 0.07	0.12 ± 0.08				
	SR(P)	0.08 ± 0.03	0.08 ± 0.03	0.09 ± 0.04				
DDMN	Traveling	0.09 ± 0.08	0.05 ± 0.06	0.10 ± 0.09			*	*
	Lingering	0.58 ± 0.06	0.63 ± 0.05	0.55 ± 0.07	***	**	***	***
	TR(A)	0.02 ± 0.02	0.01 ± 0.01	0.02 ± 0.02				
	TR(P)	0.24 ± 0.04	0.28 ± 0.05	0.23 ± 0.04	***		***	***
	SR(A)	0.02 ± 0.02	0.01 ± 0.01	0.02 ± 0.02				
	SR(P)	0.56 ± 0.07	0.62 ± 0.05	0.53 ± 0.07	***	***	***	***

Traveling scores (Equation 10) and *Lingering scores* (Equation 11) of individual participants on each group. *Traveling score* is based on the traveling rates between major basins ($TR(P)$) and their peripherals ($TR(A)$). *Lingering score* is based on the staying rates within major basins ($SR(A)$) and their peripherals ($SR(P)$).

(*) p < 0.05; (**) p < 0.01; (***) p < 0.005; between-group, pairwise comparison of traveling/lingering scores using one-way ANOVA with Bonferroni correction.

(*) p < 0.05; (**) p < 0.01; (***) p < 0.005; Kruskal-Wallis test for non-normal distribution

Table S5. Major basins transition dynamics (continued)

Network	Score	Healthy	Non-melancholic	Melancholic	p-value			
					(H,N)	(H,M)	(N,M)	KW
LN	Traveling	0.17 ± 0.11	0.15 ± 0.10	0.14 ± 0.09				
	Lingering	0.38 ± 0.07	0.48 ± 0.08	0.48 ± 0.07	***	***		***
	TR(A)	0.03 ± 0.02	0.04 ± 0.02	0.03 ± 0.02				
	TR(P)	0.20 ± 0.04	0.24 ± 0.05	0.23 ± 0.04	***	***		***
	SR(A)	0.03 ± 0.02	0.03 ± 0.02	0.03 ± 0.03				
	SR(P)	0.35 ± 0.07	0.46 ± 0.09	0.45 ± 0.08	***	***		***
LECN	Traveling	0.17 ± 0.09	0.14 ± 0.08	0.17 ± 0.08				
	Lingering	0.53 ± 0.06	0.55 ± 0.06	0.61 ± 0.06		***	***	***
	TR(A)	0.04 ± 0.02	0.04 ± 0.02	0.04 ± 0.02				
	TR(P)	0.24 ± 0.03	0.25 ± 0.04	0.26 ± 0.04		***		***
	SR(A)	0.04 ± 0.03	0.04 ± 0.03	0.04 ± 0.03				
	SR(P)	0.49 ± 0.07	0.51 ± 0.07	0.57 ± 0.07		***	***	***
PSN	Traveling	0.05 ± 0.07	0.08 ± 0.14	0.05 ± 0.07				
	Lingering	0.14 ± 0.06	0.09 ± 0.05	0.14 ± 0.07	***		***	***
	TR(A)	0.01 ± 0.01	0.01 ± 0.01	0.00 ± 0.01				
	TR(P)	0.12 ± 0.04	0.08 ± 0.02	0.10 ± 0.03	***	***	***	***
	SR(A)	0.00 ± 0.00	0.01 ± 0.01	0.00 ± 0.01	***			
	SR(P)	0.14 ± 0.06	0.09 ± 0.04	0.13 ± 0.06	***		***	***
PN	Traveling	0.83 ± 0.31	0.81 ± 0.29	0.78 ± 0.27				
	Lingering	0.29 ± 0.07	0.29 ± 0.07	0.30 ± 0.07				
	TR(A)	0.10 ± 0.03	0.09 ± 0.02	0.09 ± 0.02				
	TR(P)	0.12 ± 0.03	0.12 ± 0.03	0.13 ± 0.03				
	SR(A)	0.18 ± 0.07	0.16 ± 0.07	0.17 ± 0.08				
	SR(P)	0.11 ± 0.04	0.12 ± 0.04	0.12 ± 0.04		*		**

Traveling scores (Equation 10) and *Lingering scores* (Equation 11) of individual participants on each group. *Traveling score* is based on the traveling rates between major basins ($TR(P)$) and their peripherals ($TR(A)$). *Lingering score* is based on the staying rates within major basins ($SR(A)$) and their peripherals ($SR(P)$).

(∗) p < 0.05; (∗∗) p < 0.01; (∗∗∗) p < 0.005; between-group, pairwise comparison of traveling/lingering scores using one-way ANOVA with Bonferroni correction.

(∗) p < 0.05; (∗∗) p < 0.01; (∗∗∗) p < 0.005; Kruskal-Wallis test for non-normal distribution

Table S5. Major basins transition dynamics (continued)

Network	Score	Healthy	Non-melancholic	Melancholic	p-value			
					(H,N)	(H,M)	(N,M)	KW
RECN	Traveling	0.35 ± 0.18	0.37 ± 0.18	0.25 ± 0.11		***	***	***
	Lingering	0.18 ± 0.06	0.20 ± 0.07	0.31 ± 0.06		***	***	***
	TR(A)	0.04 ± 0.02	0.05 ± 0.02	0.04 ± 0.02				
	TR(P)	0.13 ± 0.03	0.13 ± 0.03	0.15 ± 0.03		***	***	***
	SR(A)	0.04 ± 0.03	0.04 ± 0.03	0.05 ± 0.03		*		*
	SR(P)	0.14 ± 0.05	0.16 ± 0.06	0.27 ± 0.05		***	***	***
SMN	Traveling	0.37 ± 0.15	0.35 ± 0.16	0.33 ± 0.13				
	Lingering	0.50 ± 0.06	0.49 ± 0.06	0.51 ± 0.05				
	TR(A)	0.09 ± 0.03	0.09 ± 0.03	0.08 ± 0.03				
	TR(P)	0.25 ± 0.04	0.26 ± 0.04	0.25 ± 0.04				
	SR(A)	0.13 ± 0.07	0.12 ± 0.07	0.11 ± 0.06				
	SR(P)	0.36 ± 0.09	0.38 ± 0.10	0.39 ± 0.08		*		
VDMN	Traveling	0.08 ± 0.09	0.11 ± 0.14	0.09 ± 0.10				
	Lingering	0.16 ± 0.06	0.15 ± 0.05	0.26 ± 0.07		***	***	***
	TR(A)	0.01 ± 0.01	0.01 ± 0.01	0.01 ± 0.01				
	TR(P)	0.12 ± 0.03	0.11 ± 0.03	0.14 ± 0.03		***	***	***
	SR(A)	0.01 ± 0.01	0.01 ± 0.01	0.01 ± 0.02				
	SR(P)	0.15 ± 0.06	0.14 ± 0.05	0.25 ± 0.07		***	***	***
VSPN	Traveling	0.20 ± 0.14	0.17 ± 0.13	0.17 ± 0.14				
	Lingering	0.17 ± 0.07	0.26 ± 0.07	0.22 ± 0.07	***	***	*	***
	TR(A)	0.02 ± 0.02	0.03 ± 0.02	0.02 ± 0.02				
	TR(P)	0.12 ± 0.03	0.16 ± 0.03	0.13 ± 0.03	***	**	***	***
	SR(A)	0.02 ± 0.03	0.02 ± 0.03	0.02 ± 0.03				
	SR(P)	0.14 ± 0.05	0.24 ± 0.06	0.20 ± 0.06	***	***	**	***

Traveling scores (Equation 10) and *Lingering scores* (Equation 11) of individual participants on each group. *Traveling score* is based on the traveling rates between major basins ($TR(P)$) and their peripherals ($TR(A)$). *Lingering score* is based on the staying rates within major basins ($SR(A)$) and their peripherals ($SR(P)$).

(*) $p < 0.05$; (**) $p < 0.01$; (***) $p < 0.005$; between-group, pairwise comparison of traveling/lingering scores using one-way ANOVA with Bonferroni correction.

(*) $p < 0.05$; (**) $p < 0.01$; (***) $p < 0.005$; Kruskal-Wallis test for non-normal distribution

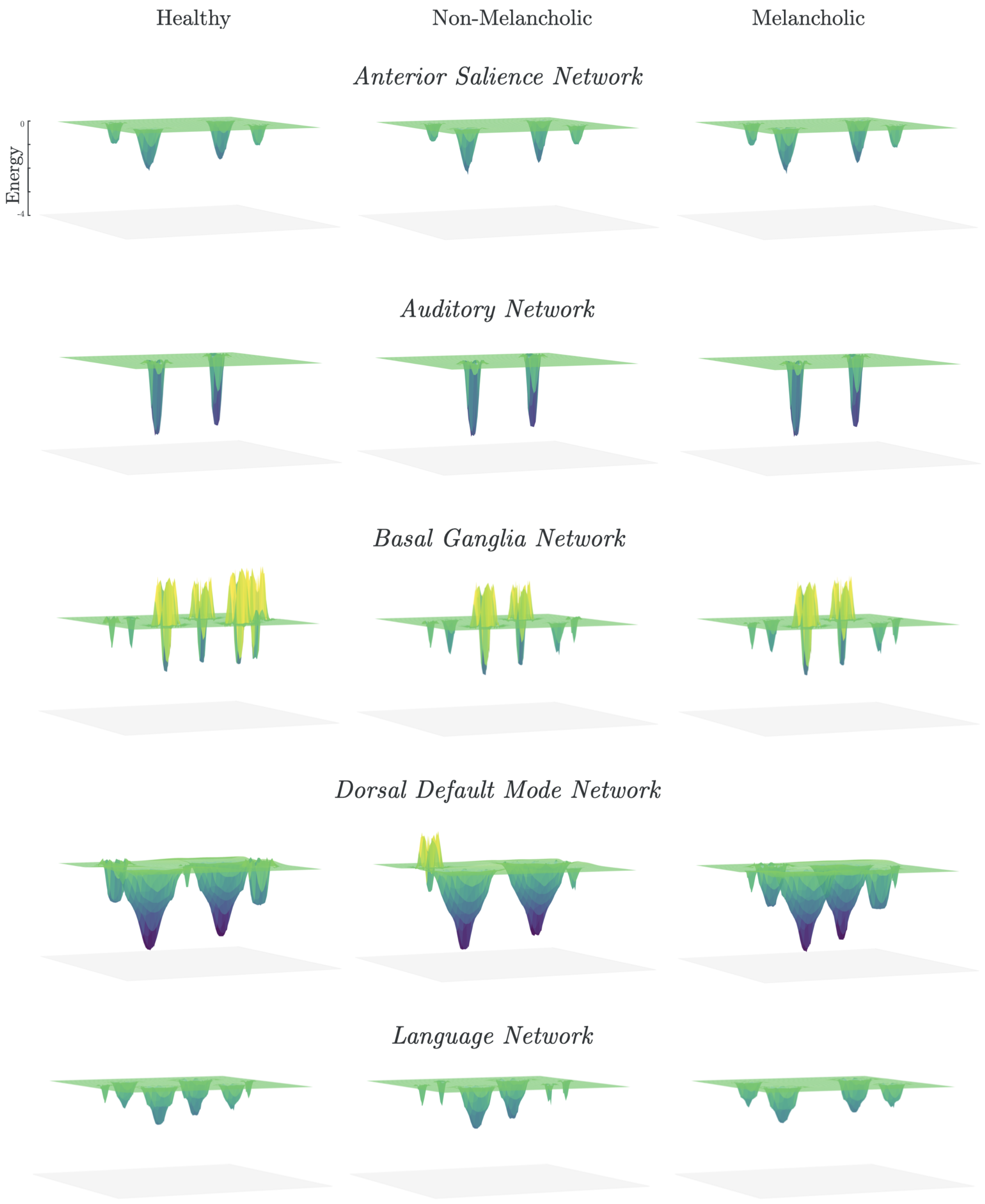

Figure S1 Energy landscapes of healthy, non-melancholic and melancholic groups

3D representations of energy landscapes across groups and networks. Basins and clusters are plotted on arbitrary state space; i.e. x- and y-locations are arbitrary. Basin sizes are proportional to the number of neighboring states clustered (Section 2.5). See Supplementary Table S3 for detailed information on individual basins.

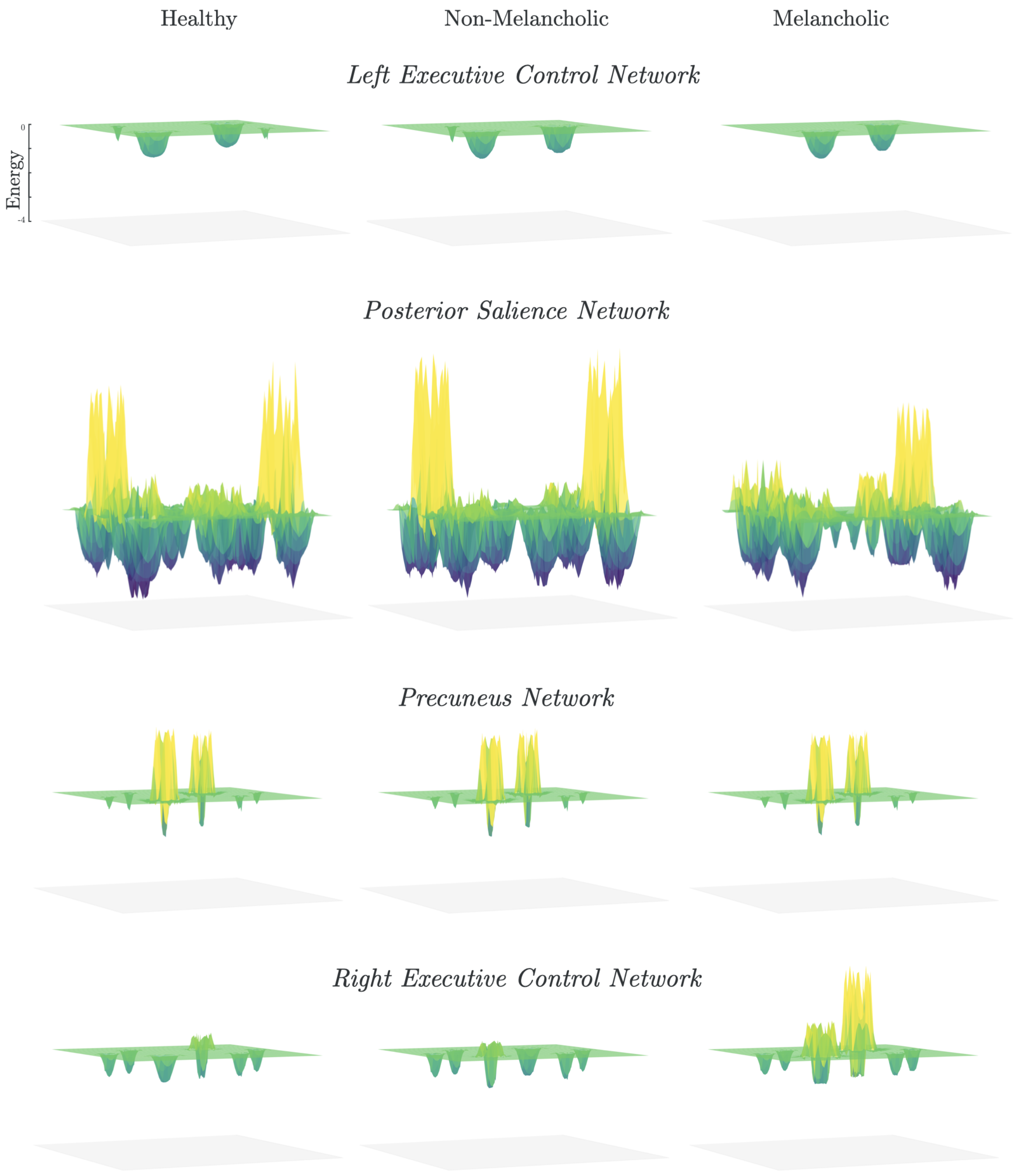

Figure S1 Energy landscapes of healthy, non-melancholic and melancholic groups (continued)

3D representations of energy landscapes across groups and networks. Basins and clusters are plotted on arbitrary state space; i.e. x- and y-locations are arbitrary. Basin sizes are proportional to the number of neighboring states clustered (Section 2.5). See Supplementary Table S3 for detailed information on individual basins.

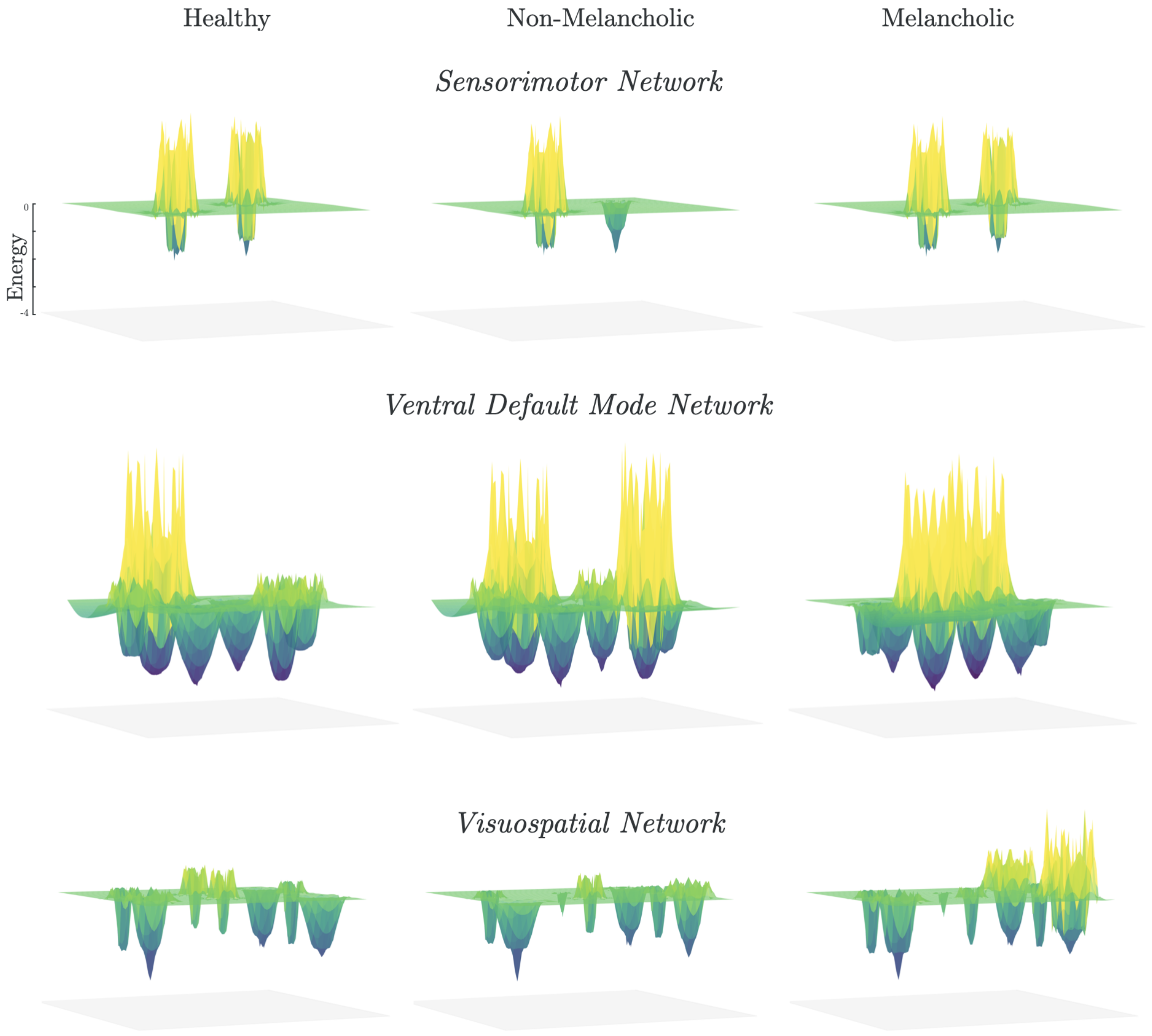

Figure S1 Energy landscapes of healthy, non-melancholic and melancholic groups (continued)

3D representations of energy landscapes across groups and networks. Basins and clusters are plotted on arbitrary state space; i.e. x- and y-locations are arbitrary. Basin sizes are proportional to the number of neighboring states clustered (Section 2.5). See Supplementary Table S3 for detailed information on individual basins.

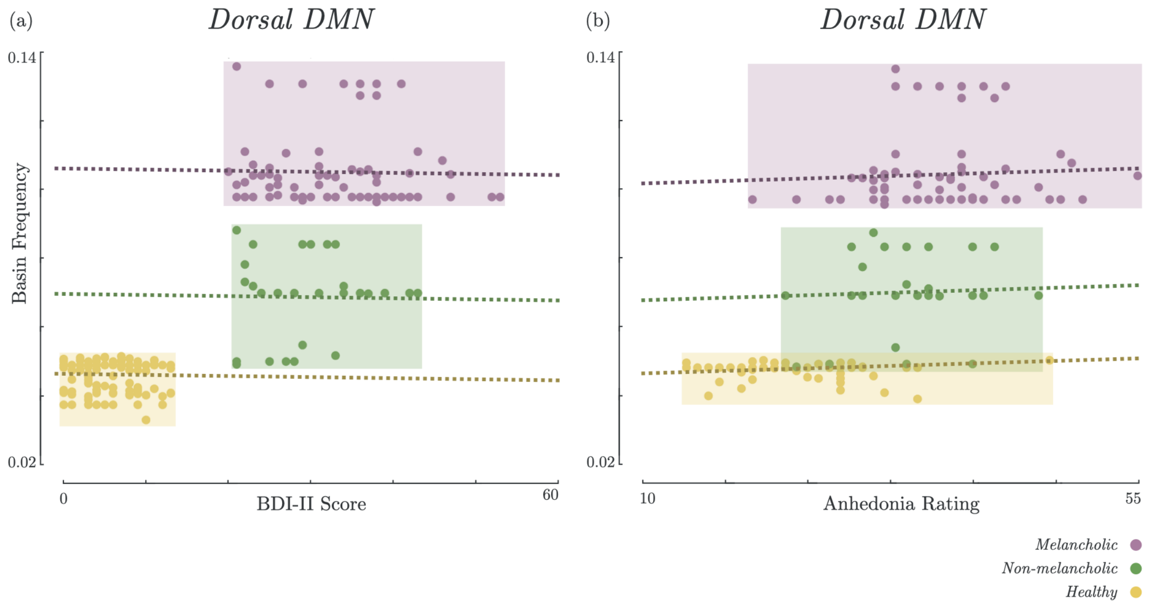

Figure S2 Correlation of basin frequencies to depressive symptoms

Correlation between average basin frequency and depressive symptom (a. depression severity; b. anhedonia) on dorsal default mode network.

Dotted lines correspond to multivariate linear regression model $y = \alpha_1 z_1 + \alpha_2 z_2 + \beta x$; y: basin frequency, x: symptom score; z_1: dummy variable for non-melancholic; z_2: dummy variable for melancholic; α, β: regression coefficients. No significant correlations between basin frequency and depression symptom (regression t-test $p_{BDI} = 0.7910$, $p_{Anhedonia} = 0.3339$).

Boxes delineate the range of data points for each group (yellow: healthy; green: non-melancholic; purple: melancholic).
